# Analysis of the association between *SLCO1B1* gene polymorphisms and coronary heart disease risk in southern Han Chinese population and statin responses in the elderly

**DOI:** 10.3389/fcvm.2026.1645446

**Published:** 2026-02-06

**Authors:** Youmin Long, Yanling Liang, Chuying Lin, Guangtie Liang, Guohao Li, Zhuoran Li, Xiuxia Lei

**Affiliations:** 1 Department of Clinical Laboratory, Guangzhou First People's Hospital, Guangzhou Medical University, Guangzhou, China; 2 Department of Clinical Laboratory, The Affiliated Foshan Women and Children Hospital, Guangdong Medical University, Foshan, China; 3 Department of Clinical Laboratory, The Sixth Affiliated Hospital, Sun Yat-sen University, Guangzhou, China; 4 Biomedical Innovation Center, The Sixth Affiliated Hospital, Sun Yat-sen University, Guangzhou, China; 5 Department of Pharmacy, Guangzhou First People's Hospital, The Second Affiliated Hospital of South China University of Technology, Guangzhou, China; 6 Department of Radiology, Guangzhou Red Cross Hospital of Jinan University, Guangzhou, China

**Keywords:** coronary heart disease, elderly population, pharmacogenetics, *SLCO1B1*, statins

## Abstract

**Objective:**

Research on *SLCO1B1* polymorphisms and statin therapy in elderly patients is limited and controversial. This study explored the association of *SLCO1B1* c.521T>C and c.388A>G variants with coronary heart disease (CHD) risk, and evaluated the lipid-lowering efficacy and safety of standard-dose statins in subjects aged >75 years to provide evidence for geriatric clinical statin use.

**Methods:**

A retrospective cohort study analyzed 6,146 individuals who were genotyped *SLCO1B1* variants c.521T>C and c.388A>G. 2318 participants were selected to evaluate the prevalence of diseases. 118 subjects (>75 years) were categorized into statin-treated (*n* = 57) and control (*n* = 61) groups. Lipid indices, liver function tests and muscle injury marker were measured by standard techniques.

**Results:**

The *SLCO1B1* c.521C allele was independently associated with increased CHD risk (odds ratio [OR] = 1.37, 95% CI:1.06–1.77, *P* = 0.02). Statin therapy significantly reduced total cholesterol (TC: 3.70 ± 0.88 vs. 4.18 ± 0.84 mmol/L, *P* = 0.004) and low-density lipoprotein cholesterol (LDL-C: 1.88 ± 0.70 vs. 2.17 ± 0.61 mmol/L, *P* = 0.04) without elevating hepatic transaminases or creatine kinase (*P* > 0.05). Carriers of the c.388A allele exhibited greater TC/LDL-C reductions compared to c.388GG homozygotes (*P* < 0.05), whereas the c.521T > C polymorphism showed no significant impact on therapeutic efficacy or safety outcomes.

**Conclusions:**

The *SLCO1B*1 c.521C allele represents an independent genetic marker for CHD susceptibility in the southern Han Chinese population. In geriatric populations, standard-dose statin therapy maintains lipid-lowering efficacy and safety, unaffected by the *SLCO1B1* c.521T>C polymorphism. Our findings provide a genetic screening tool for the primary prevention of cardiovascular diseases and offer support for the efficacy and safety of statin-based lipid-lowering therapy in adults aged >75 years.

## Introduction

Cardiovascular disease (CVD) remains the leading cause of mortality globally, accounting for 31% of annual deaths worldwide, and primarily includes coronary heart disease (CHD) (1). Statins, the cornerstone of dyslipidemia management, reduce cardiovascular events by 20%–30% through low-density lipoprotein cholesterol (LDL-C) lowering (2). However, interindividual variability in therapeutic response and adverse musculoskeletal effects, particularly in elderly populations, remain critical challenges (3). Emerging evidence implicates genetic polymorphisms in *SLCO1B1*, encoding the hepatic transporter OATP1B1, as key determinants of statin pharmacokinetics and toxicity (4). Specifically, the c.521T>C and c.388A>G variants impair OATP1B1-mediated hepatic uptake, elevating systemic statin exposure by 2–5-fold and increasing myopathy risk (5, 6). Similarly, the SLCO1B1 c.388A>G polymorphism has been consistently linked to altered OATP1B1 function, thereby affecting statin transport efficiency and potentially influencing drug exposure and clinical outcomes (7).

Despite consensus on these pharmacogenetic associations, critical knowledge gaps persist. First, while current guidelines endorse statins for primary prevention in adults aged 40–75 years (8), and a meta-analysis by Gencer et al. (9) has confirmed that statin use in individuals aged >75 years can effectively delay the progression of cardiovascular diseases and reduce the risk of mortality. However, relevant research on adverse reactions such as myopathy induced by statin administration in this population is still scarce. Additionally, existing literature (10) has verified that the SLCO1B1 genotype can guide the clinical use of statins, but evidence supporting genotype-guided dosing in patients >75 years remains sparse, with randomized controlled trials (RCTs) including <3% of this demographic (11). Second, ethnic disparities in allele frequencies—exemplified by the c.521C allele prevalence ranging from 28.3% in Amerindians to 5.7% in African-descent Brazilians and 14.8% in those of European ancestry—complicate extrapolation of existing pharmacogenetic data to admixed or understudied populations (12). Notably, no studies have systematically evaluated *SLCO1B1*-mediated statin outcomes in elderly Han Chinese.

Accordingly, this study addresses these gaps through two primary objectives: (1) to determine whether *SLCO1B1* polymorphisms independently predict CHD susceptibility in Southern Han Chinese, and (2) to evaluate genotype-dependent effects on statin efficacy and safety in this understudied demographic.

## Methods

### Study participants

This retrospective cohort study analyzed 6,146 individuals (age range: 13–101 years) receiving care at Guangzhou First People's Hospital between October 2016 and November 2018. From this population, 2,318 adults (age: 20–100 years) with complete medical records were selected for CHD prevalence analysis, comprising 1,361 males (58.71%) and 957 females (41.29%).

### CHD diagnostic criteria

Patients were classified as CHD cases if they met ≥1 of the following: Clinical diagnosis per WHO/ESC/ACC/AHA guidelines for stable/unstable angina, Coronary angiography confirmation, History of percutaneous coronary intervention (PCI).

### Cohort selection

From the CHD cohort, two age-matched groups were established:

Statin-treated group (*n* = 57): Age >75 years with at least a month of statin monotherapy; Demographic profile: 37 males (64.91%), 20 females (35.09%); mean age 84.67 ± 3.75 years

Control group (*n* = 61): Age >75 years without statin exposure; Demographic profile: 39 males (63.93%), 22 females (36.07%); mean age 83.48 ± 3.68 years.

Exclusion Criteria (applied to both groups):

1. Active systemic infections or malignancies
2. Severe hepatic/renal dysfunction (Child-Pugh C or eGFR<30 mL/min/1.73 m^2^)
3. Acute hepatitis (ALT/AST >3 × ULN)
4. Statin hypersensitivity
5. Concomitant lipid-lowering therapies (fibrates, niacin, PCSK9 inhibitors)

### Treatment protocol

Statin-treated patients were administered statins at doses within the safety range recommended by guidelines (8), see Supplementary Table S1. The therapeutic response was evaluated using laboratory data obtained 1–3 months after statin commencement.

### Ethical considerations

The study protocol was approved by the Institutional Review Boards of Guangzhou First People's Hospital. Written informed consent was obtained from all participants prior to data collection, with procedures adhering to the Declaration of Helsinki principles.

### *SLCO1B1* genotyping

Genomic DNA isolation was performed on EDTA-anticoagulated whole blood samples utilizing the DNA Blood Mini Kit (YZYMT-011, YZY Medical Technology Co., Wuhan, China), adhering to the manufacturer's spin-column protocol. Subsequent genotyping of *SLCO1B1* variants (c.521T>C and c.388A>G) was conducted via SYBR Green-based real-time PCR (YZYMT-011, YZY Medical Technology Co., Wuhan, China). The thermal cycling protocol was performed as follows: 37 ℃ for 10 min; initial denaturation at 95 ℃ for 5 min; followed by 40 amplification cycles consisting of denaturation at 95 ℃ for 15 s and combined annealing/extension at 60 ℃ for 1 min. Fluorescence signals were collected as FAM channel (c.388A; c.521 T) and VIC channel (c.388G, c.521C) and ROX channel (internal reference).

### Laboratory assessments

Serum biochemical parameters including hepatic enzymes [alanine aminotransferase (ALT), aspartate aminotransferase (AST)], creatine kinase (CK), and lipid profile components [triglycerides (TG), total cholesterol (TC), high-density lipoprotein cholesterol (HDL-C), low-density lipoprotein cholesterol (LDL-C)] were quantified using a Beckman Coulter AU5800 clinical chemistry analyzer (Beckman Coulter Inc., Brea, CA, USA) with standardized enzymatic assays. For the statin-treated cohort, follow-up measurements of these biomarkers were performed 4–12 weeks after treatment initiation to assess drug-related metabolic changes.

### Statistical analysis

All analyses were conducted using SPSS Statistics version 17.0 (IBM Corp., Armonk, NY, USA). Continuous variables are presented as mean ± standard deviation (SD). Between-group comparisons of baseline data (statin-treated vs. control) were performed using independent samples t-tests following verification of normality (Shapiro–Wilk test) and homogeneity of variance (Levene's test). One-way analysis of covariance was applied to compare differences in biochemical index levels between the post-medication statin-treated group and the control group. The chi-square test was used to analyze and compare the baseline levels of different *SLCO1B1* genotypes in the statin-treated group. Non-normally distributed variables were analyzed via the Kruskal–Wallis H test to assess genotype-phenotype correlations between *SLCO1B1* variants and percentage changes in biochemical parameters. Multivariable binary logistic regression models were constructed to identify coronary heart disease risk factors, with adjustment for age, sex, and baseline lipid levels. Results are reported as adjusted odds ratios (OR) with 95% confidence intervals (CI), and visualized using R packages (forestplot package). Population genetic structure was evaluated through Hardy-Weinberg equilibrium testing using Pearson's *χ*^2^ test (*α* = 0.05). A two-tailed *α* level of 0.05 defined statistical significance. Effect sizes were calculated for significant findings [Cohen's d for t-tests; eta-squared (*η*^2^) for ANOVA analogues]. A covariance analysis model was constructed with the percentage changes in low-density lipoprotein cholesterol (LDL-C) and creatine kinase (CK) as the dependent variables. We incorporated the multiplicative interaction term between sex and genotype into the model, which was used to examine the association between *SLCO1B1* genotypes and statin efficacy/safety across different sexes. All interaction models were adjusted for predefined covariates, including age, baseline LDL-C and CK and levels. A *P*-value < 0.05 was considered statistically significant.

## Results

### Genetic equilibrium of *SLCO1B1* variants

Both *SLCO1B1* variants (c.521T>C and c.388A>G) exhibited genotype distributions consistent with Hardy-Weinberg equilibrium in the cohort (*P* = 0.13 and *P* = 0.89, respectively). This confirms appropriate population genetic structure for subsequent association studies (Table 1).

**Table 1:** Hardy-Weinberg equilibrium analysis of *SLCO1B1* variants (c.521T>C and c.388A>G).

Variant	Genotype	Observed count	Expected count	*χ*^2^ contribution	Total*χ*^2^ (df = 1)	*P*-value
c.521T>C *N* = 6,146	TT	4,701	4,713.85	0.035	2.27	0.13
	TC	1,363	1,337.31	0.485		
	CC	82	94.84	1.754		
c.388A>G *N* = 6,146	AA	346	343.98	0.012	0.02	0.89
	AG	2,216	2,220.04	0.007		
	GG	3,584	3,581.98	0.001		

*SLCO1B1*, solute carrier organic anion transporter 1B1; SNP, single-nucleotide polymorphism; *N*, number of subjects.

### Identification of multifactorial determinants for CHD

**Figure 1:**
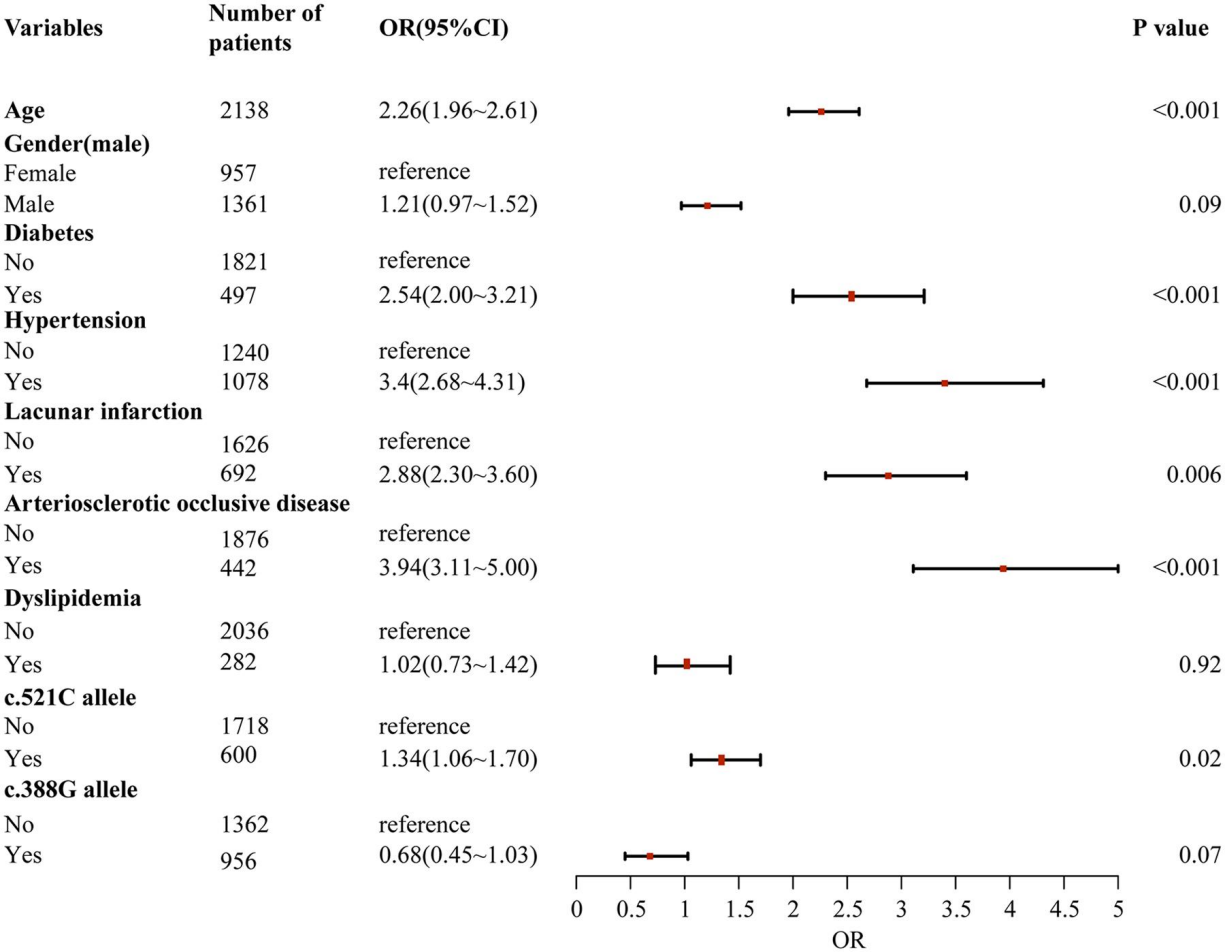
Univariate logistic regression analysis of risk factors for coronary heart disease in southern Han Chinese population.

**Figure 2:**
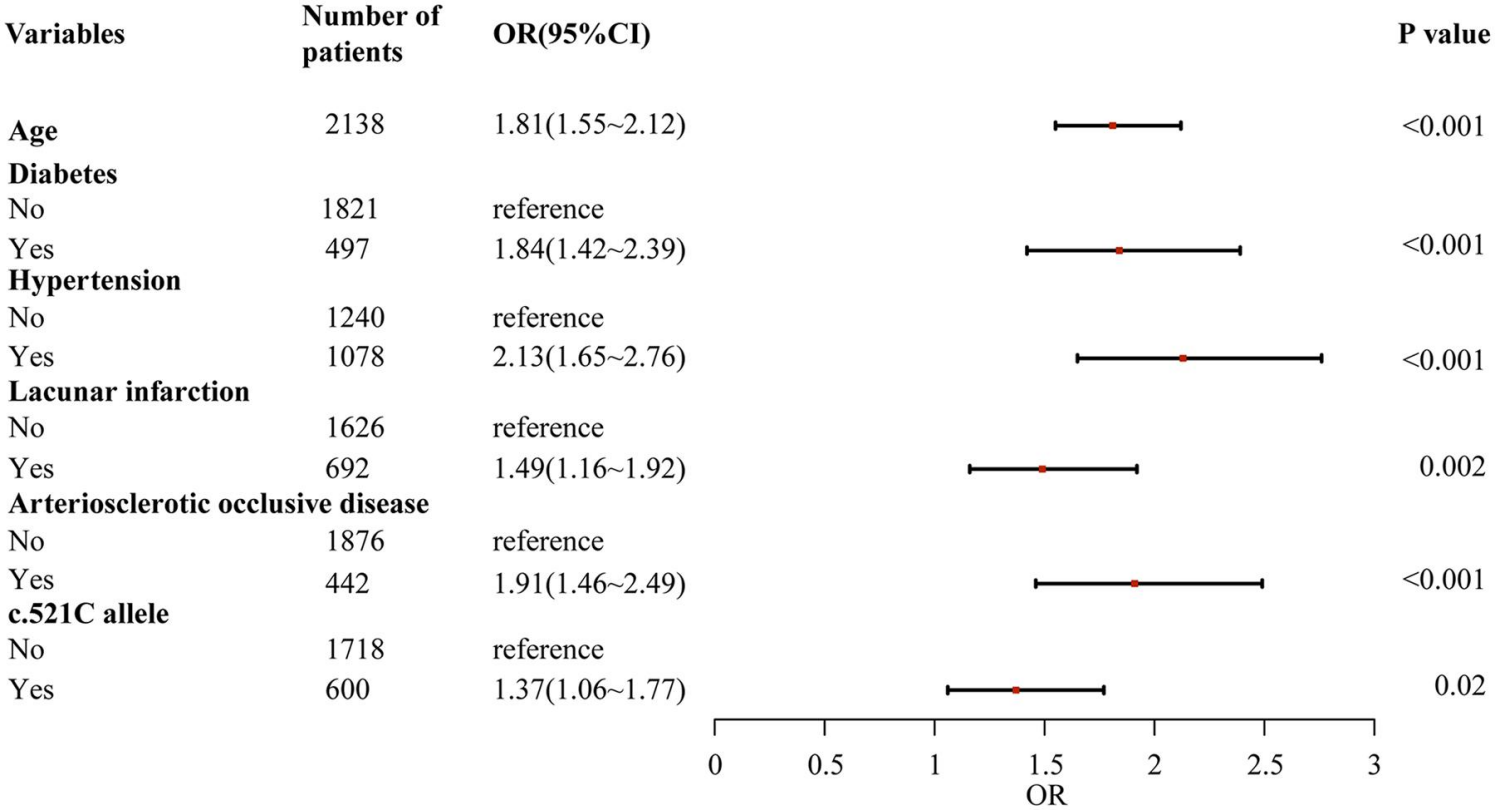
Multiple logistic regression analysis of risk factors for coronary heart disease in southern Han Chinese population.

Through a two-stage logistic regression analysis evaluating CHD determinants in Southern Chinese Han individuals, univariate screening identified candidate risk factors subsequently analyzed via multivariable models adjusted for age, sex, and baseline lipid profiles. The final analysis demonstrated independent associations between CHD incidence and advancing age [odds ratio (OR) = 1.08 per year, 95% confidence interval (CI): 1.03–1.13], diabetes (OR = 2.41, 95% CI:1.76–3.29), hypertension (OR = 1.92, 95% CI:1.34–2.75), lacunar infarction (OR = 1.67, 95% CI:1.21–2.31), arteriosclerotic occlusion (OR = 2.15, 95% CI:1.52–3.04), and the *SLCO1B1* c.521C allele (OR = 1.37, 95% CI:1.06–1.77, *P* = 0.02), collectively underscoring the multifactorial etiology integrating conventional cardiometabolic risks and genetic susceptibility in this population (Figures 1, 2).

### Baseline characteristics of study cohorts

Table 2 summarizes the clinical profiles of the statin-treated and control groups, including demographic characteristics (sex, age), lipid parameters (TG, TC, HDL-C, LDL-C), muscle/hepatic biomarkers (CK, ALT, AST), and comorbidities (hypertension, CHD, diabetes, dyslipidemiaitalic). Baseline variables were balanced between groups (all *P* > 0.05), with the exception of diabetes prevalence (*P* = 0.03). Since diabetes can affect lipid metabolism, the comparison between the two groups in the subsequent analysis will be adjusted for diabetes using one-way analysis of covariance.

**Table 2:** Baseline characteristics of statin-treated and control cohorts in elderly patients.

Variable	Statin-treated group (*n* = 57)	Control group (*n* = 61)	*P*-value
Demographic			
Male sex, *n* (%)	37 (64.9%)	39 (63.9%)	0.91
Age (years)	84.67 ± 3.75	83.48 ± 3.68	0.08
Lipid profile			
TC (mmol/L)	4.13 ± 1.06	4.18 ± 0.84	0.81
LDL-C (mmol/L)	2.17 ± 0.78	2.17 ± 0.61	0.98
HDL-C (mmol/L)	1.13 ± 0.26	1.13 ± 0.30	0.97
TG (mmol/L)	1.36 ± 0.70	1.29 ± 1.11	0.65
Safety biomarkers			
CK (U/L)	96.37 ± 82.91	100.61 ± 102.34	0.81
ALT (U/L)	16.58 ± 12.56	16.07 ± 11.20	0.81
AST (U/L)	25.46 ± 21.49	22.67 ± 15.87	0.42
Comorbidities			
Hypertension, *n* (%)	49 (85.96%)	48 (78.69%)	0.30
Diabetes, *n* (%)	24 (42.11%)	14 (22.95%)	**0**.**03**
CHD, *n* (%)	31 (54.39%)	26 (42.62%)	0.20
Dyslipidemia, *n* (%)	10 (17.54%)	10 (16.39%)	0.87

TC, total cholesterol; TG, Triglycerides; LDL-C, low-density lipoprotein cholesterol; HDL-C, high-density lipoprotein cholesterol; CK, Creatine kinase; ALT, alanine aminotransferase; AST, aspartate aminotransferase; CHD, Coronary heart disease; *n*, number of subjects.

Bold values indicate statistical significance (*P* < 0.05).

### Efficacy and safety of statin therapy in adults aged >75 years

Comparative analysis of post-intervention biomarkers demonstrated statistically significant attenuation of TC (3.70 ± 0.88 vs. 4.18 ± 0.84 mmol/L; *P* = 0.004) and LDL-C (1.88 ± 0.70 vs. 2.17 ± 0.61 mmol/L; *P* = 0.04) in the statin cohort relative to untreated controls (Table 3) No statistically significant intergroup differences were observed for HDL-C, TG, ALT, AST, or CK levels (all *P* > 0.05).

**Table 3:** Metabolic and safety outcomes after statin therapy in elderly patients.

Variable	Statin-treated group (*n* = 57)	Control group (*n* = 61)	*P*-value
Lipid profile			
TC (mmol/L)	3.70 ± 0.88	4.18 ± 0.84	**0**.**004**
LDL-C (mmol/L)	1.88 ± 0.70	2.17 ± 0.61	**0**.**04**
HDL-C (mmol/L)	1.09 ± 0.29	1.13 ± 0.30	0.92
TG (mmol/L)	1.33 ± 0.79	1.29 ± 1.11	0.54
Safety biomarkers			
CK (U/L)	78.72 ± 62.01	100.61 ± 102.34	0.14
ALT (U/L)	21.14 ± 28.00	16.07 ± 11.20	0.29
AST (U/L)	23.27 ± 16.50	22.67 ± 15.87	0.89

TC, total cholesterol; TG, Triglycerides; LDL-C, low-density lipoprotein cholesterol; HDL-C, high-density lipoprotein cholesterol; CK, Creatine kinase; ALT, alanine aminotransferase; AST, aspartate aminotransferase; *n*, number of subjects.

Bold values indicate statistical significance (*P* < 0.05).

### Impact of *SLCO1B1* polymorphisms on statin response in elderly patients

Genetic stratification analysis (Tables 4, 5) demonstrated that carriers of the *SLCO1B1* c.388A allele exhibited significantly greater reductions in TC and LDL-C compared to c.388GG homozygotes (*P* < 0.05). However, Kruskal–Wallis tests revealed no genoty*p*e-dependent differences in HDL-C, TG, ALT, AST, or CK changes across *SLCO1B1* c.521T>C or c.388A>G variants (all *P* > 0.05).

**Table 4:** Differential responses in lipid profiles biomarkers across *SLCO1B1* genotypes after statin therapy in elderly patients.

Variable	Genotype	Baseline (mmol/L)	After statins (mmol/L)	Mean % change (95% CI)	*P* value
TC	CC (13)	4.44 ± 0.57	4.16 ± 0.73	−5.50 (−15.06, 4.05)	–
	TC (24)	4.12 ± 1.13	3.51 ± 0.71	−11.00 (−19.86, −2.16)	0.78
	TT (20)	3.96 ± 1.22	3.62 ± 1.06	−4.62 (−18.28, 9.05)	–
	AA (3)	5.04 ± 2.26	4.22 ± 1.09	−10.46 (−63.41, 42.48)	–
	AG (16)	4.34 ± 0.96	3.38 ± 0.57	−19.38 (−29.08, −9.67)	**0.04**
	GG (38)	3.98 ± 0.98	3.79 ± 0.94	−2.28 (−10.04, 5.48)	–
LDL-C	CC (13)	2.42 ± 0.57	2.30 ± 0.77	−4.66 (−18.42, 9.09)	–
	TC (24)	2.17 ± 0.77	1.71 ± 0.52	−15.43 (−27.64, −3.22)	0.38
	TT (20)	2.00 ± 0.90	1.81 ± 0.75	−0.60 (−21.38, 20.17)	–
	AA (3)	2.92 ± 1.59	2.42 ± 0.97	−10.77 (−58.97, 37.42)	–
	AG (16)	2.33 ± 0.63	1.61 ± 0.40	−27.16 (−39.24, −15.07)	**0.01**
	GG (38)	2.04 ± 0.74	1.95 ± 0.75	0.63 (−11.24, 12.50)	–
HDL-C	CC (13)	1.24 ± 0.28	1.20 ± 0.26	−2.11 (−10.32, 6.11)	–
	TC (24)	1.05 ± 0.18	1.04 ± 0.30	0.36 (−13.76, 14.48)	0.99
	TT (20)	1.14 ± 0.31	1.09 ± 0.28	−2.44 (−11.30, 6.42)	–
	AA (3)	1.24 ± 0.50	1.15 ± 0.29	−2.54 (−52.90, 47.81)	–
	AG (16)	1.13 ± 0.18	1.08 ± 0.22	−3.35 (−13.40, 6.70)	0.95
	GG (38)	1.12 ± 0.27	1.09 ± 0.32	−0.17 (−9.30, 8.96)	–
TG	CC (13)	1.22 ± 0.56	1.31 ± 0.61	10.49 (−6.07, 27.05)	–
	TC (24)	1.43 ± 0.67	1.37 ± 0.97	1.85 (−20.05, 23.75)	0.49
	TT (20)	1.38 ± 0.83	1.29 ± 0.69	4.65 (−18.00, 27.29)	–
	AA (3)	1.17 ± 0.29	1.05 ± 0.38	−11.42 (−74.48, 51.64)	–
	AG (16)	1.34 ± 0.80	1.40 ± 1.03	13.74 (−15.03, 42.51)	0.70
	GG (38)	1.39 ± 0.69	1.32 ± 0.71	2.32 (−11.85, 16.48)	–

TC, total cholesterol; LDL-C, low-density lipoprotein cholesterol; HDL-C, high-density lipoprotein cholesterol; TG, triglyceride; CI, confidence interval.

Bold values indicate statistical significance (*P* < 0.05).

**Table 5:** Differential responses in hepatic/muscular biomarkers across *SLCO1B1* genotypes after statin therapy in elderly patients.

Variable	Genotype	Baseline (U/L)	After statins (U/L)	Mean % change (95% CI)	*P* value
AST	CC (13)	26.07 ± 21.63	28.08 ± 31.08	76.91 (−55.23, 209.05)	–
	TC (24)	22.05 ± 7.51	23.86 ± 10.26	19.15 (−8.14, 46.45)	0.20
	TT (20)	29.15 ± 31.11	19.45 ± 5.79	−8.55 (−28.19, 11.10)	–
	AA (3)	19.00 ± 2.65	22.00 ± 2.65	16.21 (−8.89, 41.32)	–
	AG (16)	20.28 ± 8.08	22.00 ± 7.36	17.62 (−8.71, 43.96)	0.24
	GG (38)	28.15 ± 25.49	23.91 ± 19.71	25.21 (−20.76, 71.18)	–
ALT	CC (13)	18.74 ± 11.67	33.28 ± 54.08	98.31 (−82.38, 279.01)	–
	TC (24)	15.18 ± 9.76	21.13 ± 13.95	127.81 (−36.39, 292.02)	0.13
	TT (20)	16.85 ± 16.06	13.24 ± 7.77	16.34 (−27.17, 59.85)	–
	AA (3)	14.00 ± 6.56	17.00 ± 9.54	17.22 (−20.44, 54.88)	–
	AG (16)	14.24 ± 10.21	21.31 ± 11.84	185.70 (−63.20, 434.62)	0.11
	GG (38)	17.77 ± 13.78	21.39 ± 33.51	43.40 (−19.17, 105.96)	–
CK	CC (13)	73.41 ± 42.24	66.00 ± 39.68	−1.18 (−28.01, 25.66)	–
	TC (24)	112.63 ± 94.00	93.75 ± 67.21	36.20 (−23.23, 95.64)	0.40
	TT (20)	91.80 ± 87.87	68.95 ± 66.14	−5.86 (−49.63, 37.91)	–
	AA (3)	72.67 ± 43.78	75.00 ± 48.12	0.55 (−20.83, 21.92)	–
	AG (16)	115.50 ± 82.07	93.63 ± 57.61	22.76 (−56.89, 102.41)	0.54
	GG (38)	90.19 ± 85.60	72.74 ± 64.92	9.75 (−21.32, 40.82)	–

ALT, alanine aminotransferase; AST, aspartate aminotransferase; CK, creatine kinase; CI, confidence interval.

### Exploratory analysis of sex-specific effects of *SLCO1B1* polymorphisms on statin efficacy and adverse reactions

Supplementary Table S2 summarizes the clinical profiles of the statin-treated group, stratified by sex. To further investigate whether sex modifies the association between *SLCO1B1* genotype and statin efficacy as well as adverse reactions, we conducted an exploratory analysis by incorporating an interaction term between sex and *SLCO1B1* c.521T>C polymorphism into the regression model. The results (Tables 6, 7) demonstrated that the interaction between sex and *SLCO1B1* c.521T>C genotype on LDL-C reduction did not reach statistical significance (*P* for interaction = 0.22). Similarly, no statistically significant interaction was detected with respect to CK elevation (*P* for interaction = 0.88).

**Table 6:** Interaction between sex and *SLCO1B1* c.521T>C variants on statin-induced LDL-C lowering efficacy.

Subgroup	*n*	LDL-C, mean % change (95% CI)	Adjusted mean difference (95% CI)^a^	*P* value	*P* for interaction*
Overall	57	−7.77 (−16.84, 1.30)	−5.56 (−23.61, 12.49)	0.54	–
By sex					0.22
Male	37	−6.72 (−17.82, 4.39)	0.78 (−21.02, 22.58)	0.94	
Female	20	−9.72 (26.83, 7.39)	−29.37 (−76.34, 17.59)	0.20	
By genotype					
TT (Reference)	20	−0.60 (21.38, 20.17)	Ref		
TC/CC	37	−11.65 (−20.69, −2.61)	−5.41 (−22.76, 11.95)	0.54	

a Mean differences were derived from an analysis of covariance (ANCOVA) model adjusting for age and baseline LDL-C.

* The *P* for interaction was tested by including a sex genotype term in the model.

**Table 7:** Effect of sex on the association between *SLCO1B1* c.521T>C variants and creatine kinase elevation during statin therapy.

Subgroup	*n*	CK, mean % change (95% CI)	Adjusted mean difference (95% CI)^a^	*P* value	*P* for interaction*
Overall	57	12.92 (−16.07, 41.90)	31.22 (−26.93, 89.36)	0.29	–
By sex					0.88
Male	37	27.80 (−16.01, 71.61)	44.31 (−40.80, 129.41)	0.30	
Female	20	−14.61 (31.01, 1.79)	42.78 (7.84, 77.72)	**0**.**02**	
By genotype					
TT (Reference)	20	−5.86 (−49.64, 37.91)	Ref		
TC/CC	37	23.07 (−15.88, 62.01)	−5.41 (−22.76, 11.95)	0.54	

a Mean differences were derived from an analysis of covariance (ANCOVA) model adjusting for age and baseline CK.

* The *P* for interaction was tested by including a sex genotype term in the model.

Bold values indicate statistical significance (*P* < 0.05).

## Discussion

This study demonstrates that age, diabetes, and hypertension act as independent genetic risk factors for CHD in the southern Han Chinese population, with findings congruent with established CHD risk markers and providing robust validation of prior epidemiological evidence (13). Notably, our research further identifies the *SLCO1B1* c.521C allele as a distinct genetic determinant for CHD in this cohort (OR = 1.37, 95% CI: 1.06–1.77, *P* = 0.02), which is consistent with investigations by MO, Q et al (14). As a transmembrane transporter, OATP1B1 mediates hepatic uptake and clearance of endogenous substrates including bilirubin and bile acids. Clinical study (15) have postulated that the *SLCO1B1* 521C allele induces structural alterations in OATP1B1, leading to diminished substrate binding affinity and reduced transport efficiency. This functional impairment compromises hepatic processing of lipophilic molecules, exacerbating dyslipidemia through mechanisms such as increased retention of triglyceride-rich lipoproteins and reduced reverse cholesterol transport. Consequently, the c.521C allele-driven dysfunction may establish a genetic predisposition to CHD by promoting atherogenic lipid profiles and vascular inflammation. In this context, implementing time-sensitive primary prevention strategies—such as intensified lipid-lowering therapy and lifestyle interventions—for individuals harboring the c.521C allele may counteract the genetic risk and attenuate disease incidence and progression.

In the field of CHD prevention and management, strategies predominantly encompass lifestyle modifications and lipid-lowering interventions. Within the domain of dyslipidemia therapy, low-density lipoprotein cholesterol (LDL-C) has been firmly established as a pivotal risk factor for CHD, with robust evidence demonstrating that lower LDL-C concentrations are inversely associated with reduced incidence and mortality from cardiovascular events (16). A meta-analytic synthesis (17) has underscored that each 1 mmol/L (38.7 mg/dL) reduction in LDL-C is accompanied by a 22% relative risk reduction in major vascular and coronary artery events. These data reinforce the indispensable role of achieving and maintaining low LDL-C levels in effectively mitigating the initiation and progression of CHD pathogenesis. Since the advent of statins, these agents have emerged as the clinical gold standard for lipid-lowering therapy owing to their hypolipidemic efficacy, being widely deployed in the management of dyslipidemia and primary/secondary prevention of CHD (18, 19). Notwithstanding, the clinical applicability of statins in geriatric populations remains a subject of debate due to potential side effects such as myotoxicity. Although meta-analyses have confirmed that statin use in elderly individuals aged ≥75 years can effectively reduce the incidence of cardiovascular diseases (9), evidence regarding the safety of such drugs in this specific population remains insufficient. Geriatric individuals constitute a distinct therapeutic cohort with age-related metabolic derangements that diverge significantly from younger populations, rendering their pharmacotherapeutic management susceptible to multifactorial influences. Therefore, our investigation was designed to focus on geriatric patients with age >75 years.

Our findings demonstrate that statin therapy in this demographic predominantly elicits reductions in plasma TC and LDL-C concentrations, with negligible effects on HDL-C and TG. These data suggest that the use of statins in adults aged over 75 years can effectively reduce LDL and TC, thereby preventing and treating CHD. Additionally, we found that routine doses of statins in older adults did not provoke severe hepatotoxicity (aspartate transaminase/alanine transaminase elevation: *P* > 0.05) or myopathic complications (creatine kinase levels: *P* > 0.05). These safety observations are consistent with dose-dependent hepatotoxicity patterns and the low adverse event rates reported for standard/low-dose regimens (20, 21), suggesting that statin use at routine or low doses is safe in elderly.

With the continuous advancement of pharmacogenomics, personalized medication has garnered increasing attention. Previous studies have demonstrated that the *SLCO1B1* gene influences the hepatic transport of statins by encoding OATP1B1, thereby affecting their pharmacological efficacy. However, current conclusions regarding the impact of *SLCO1B1* gene polymorphisms on the lipid-modifying effects and adverse reactions of statins remain inconsistent. Our study revealed that the c.521T>C polymorphism in the *SLCO1B1* gene had no significant effect on the lipid-lowering efficacy of statins in older adults, with no significant differences in the percentage changes of blood lipid levels across genotypes (*P* > 0.05). These results are consistent with those reported by Kim Y et al (22). Additionally, statins exhibited more pronounced lipid-lowering effects in patients carrying the c.388A allele. In contrast, Wu et al. found that individuals with the c.388G allele showed greater reductions in total cholesterol (TC) and low-density lipoprotein cholesterol (LDL-C) after 4 weeks of simvastatin treatment (23), which is inconsistent with the findings of our study. The reasons for this are unclear but we hypothesize that this discrepancy could be attributed to the relatively small sample size of patients harboring the c.388A allele in our study. Notably, a recent study has proposed that c.521T>C is unrelated to statin-induced myotoxicity side effects (24). Our study further demonstrates that neither the *SLCO1B1* c.521T>C nor c.388A>G polymorphisms exert significant effects on hepatic function markers (AST, ALT) or muscular safety parameters (CK) in elderly patients (all *P* > 0.05). These findings substantiate the reliable safety profile of standard-dose statin therapy across diverse *SLCO1B1* genotypes in elderly populations. Furthermore, no statistically significant sex-specific differences were observed in the association between the *SLCO1B1* c.521T>C genotype and statin therapeutic response or adverse outcomes in this study (all interaction *P* > 0.05). This finding diverges from earlier reports such as that by Bosco et al (25), which suggested that the genotype effect of SLCO1B1 rs4149056 could be more pronounced in women than men. Several factors may account for this discrepancy. First, population characteristics differed: our cohort was restricted to older adults (age > 75 years), whereas the study by Bosco et al. included predominantly younger participants. Age-related physiological changes—including alterations in hepatic and renal function, sex-hormone profiles, and body composition—could mask or modify sexually dimorphic pharmacogenetic relationships. Second, limitations in statistical power must be acknowledged; the relatively small sample size of the statin-treated group (*n* = 57) reduces the ability to detect modest gene-by-sex interactions, thereby increasing the risk of a type II error. In summary, while our results provide preliminary support for a sex-independent effect of the *SLCO1B1* c.521T>C variant in an elderly population, they also underscore the need for future prospective, adequately powered studies conducted in well-defined geriatric cohorts under uniform treatment protocols to clarify the complex interplay of sex and age in this pharmacogenetic association.

In summary, this paper represents the *SLCO1B1* c.521C allele as a novel genetic risk marker for CHD while affirming the lipid-lowering efficacy and safety of conventional statin regimens in elderly populations. *SLCO1B1* genotyping may hold limited utility for personalizing statin therapy in older adults receiving standard doses, and such genotyping may be unnecessary in this population. These findings provide valuable insights for optimizing cardiovascular risk management in geriatric populations through genetic profiling and personalized statin regimens.

## Study limitations

Notably, as a retrospective study, there are several limitations to our study. First, this study focuses on the Han Chinese population in South China, and its applicability to other ethnic groups requires further research and validation. Second, the small sample size of the elderly subgroup may reduce the power to detect small effect sizes, thereby reducing the likelihood of obtaining conclusive results. In particular, the low allele frequency of c.388A, which makes the observed enhanced lipid-lowering effect associated with this allele may be coincidental. Third, the present investigation adopted a surrogate marker-driven paradigm for both efficacy and safety assessments. While this approach permits precise quantification of pharmacological effects, it inherently fails to address the clinically paramount question of whether observed biochemical improvements translate into reduced cardiovascular event rates, nor does it adequately reflect treatment tolerability from the patient perspective. Forth, although the interaction analysis did not demonstrate statistical significance, the overall sample size of the treatment group (*n* = 57) was limited, and the subgroup sizes became even smaller after cross-stratification by sex and genotype, resulting in insufficient statistical power to detect gene-by-sex interactions. Consequently, conclusions regarding sex differences should be considered exploratory. Despite these limitations, the present study still identifies SLCO1B1 c.521C as a genetically meaningful marker with clinical utility for cardiovascular disease (CVD) prevention in southern Han Chinese population. It also confirms that the efficacy of statins in elderly individuals aged over 75 years is independent of genotype, which suggests that the need for pharmacogenetic testing in statin therapy for elderly patients is limited.

## Data Availability

The raw data supporting the conclusions of this article will be made available by the authors, without undue reservation.
