# Supplementary material for: Analysis of the association between SLCO1B1 gene polymorphisms and coronary heart disease risk in southern Han Chinese population and statin responses in the elderly
Source: Front Cardiovasc Med. 2026 Feb 6;13:1645446. doi: 10.3389/fcvm.2026.1645446 (PMC12920528; doi:10.3389/fcvm.2026.1645446)
Supplement: Supplementary file 1 [file Datasheet1.docx]

**Supplementary Table 1. Types and dosage of statins in the statin-treated group**

| Atorvastatin | Fluvastatin | Pitavastatin | Pravastatin | Rosuvastatin | Simvastatin |
| --- | --- | --- | --- | --- | --- |
| 10 mg/d | 80 mg/d | 2 mg/d | 0.5 mg/d | 2 mg/d | 10 mg/d |
| 20 mg/d | ------ | ------ | 20 mg/d | 5 mg/d | 20 mg/d |
| ------ | ------ | ------ | 40 mg/d | 10 mg/d | 40 mg/d |
| ------ | ------ | ------ | ------ | 20 mg/d |  |

**Supplementary Table 2. Baseline Characteristics of the Statin-Treated group, Overall and Stratified by Sex**

| **Variable** | **Overall (n=57)** | **Stratified by Sex** | |  |
| --- | --- | --- | --- | --- |
|  |  | **Male**  **(n=37)** | **Female**  **(n=20)** | ***P* value** |
| **Demographic** |  |  |  |  |
| Age (years) | 84.67 ± 3.75 | 84.05 ± 3.67 | 85.8 ± 3.72 | 0.10 |
| **Clinical & Labs** |  |  |  |  |
| LDL-C (mmol/L) | 2.17 ± 0.78 | 2.10 ± 0.74 | 2.30 ± 0.86 | 0.36 |
| CK (U/L) | 96.37 ± 82.91 | 102.46 ± 87.86 | 85.12 ± 73.69 | 0.46 |
| Hypertension, n (%) | 49 (85.96%) | 32 (86.5%) | 17 (85.0%) | 0.59 |
| Diabetes, n (%) | 24 (42.11%) | 16 (43.2%) | 8 (40.0%) | 0.52 |
| CHD, n (%) | 31 (54.39%) | 23 (62.2%) | 8 (40.0%) | 0.09 |
| Dyslipidemia, n (%) | 10 (17.54%) | 5 (13.5%) | 5 (25.0%) | 0.23 |
| **Genetic** |  |  |  |  |
| SLCO1B1 c.521C allele frequency, n (%) | 37 (64.9%) | 21 (56.8%) | 16 (80%) | 0.09 |
